# Characteristics of and Risk Factors for Migration of Biliary Plastic Stents After Stone Removal With Endoscopic Retrograde Cholangiopancreatography

**DOI:** 10.1155/cjgh/9996501

**Published:** 2025-09-03

**Authors:** Longping Chen, Yanfang Lin, Linfu Zheng, Chuanshen Jiang, Zhiping Chen, Jin Zheng, Dazhou Li, Wen Wang

**Affiliations:** ^1^ Department of Gastroenterology, Fuzong Clinical Medical College of Fujian Medical University, Fuzhou, 350025, China, fjmu.edu.cn; ^2^ Department of Gastroenterology, 900th Hospital of PLA Joint Logistic Support Force, Fuzhou, 350025, China; ^3^ Department of Gastroenterology, Dongfang Hospital of Xiamen University, Fuzhou, 350025, China

**Keywords:** biliary plastic stent, endoscopic retrograde cholangiopancreatography, risk factors, stent migration

## Abstract

**Background and Aims:** Biliary plastic stents frequently detach or migrate from the duodenal papilla, leading to recurrent biliary tract infections or intestinal perforations. This study aimed to investigate the incidence and risk factors associated with migration of biliary plastic stents following endoscopic retrograde cholangiopancreatography (ERCP).

**Methods:** The study analyzed a population of patients who underwent ERCP for bile duct stone removal and placement of biliary plastic stents between March 2018 and December 2022, and analyzed the risk factors for biliary stent migration in the enrolled patients.

**Results:** A total of 836 patients (60.40 ± 15.58 years, 511 males) were included in the analysis. Among them, 105 patients experienced stent migration, comprising 91 (10.9%) patients with distal biliary plastic stent migration and 14 (1.7%) patients with proximal biliary plastic stent migration. Multivariate logistic regression analysis revealed that the risk of stent migration was significantly higher in patients with duodenal diverticula (odds ratio [OR] = 2.367, *p* = 0.005), duodenal sphincter incision (OR = 3.638, *p* = 0.007), and longer stent lengths (OR = 0.423, *p* < 0.001). The Christmas tree plastic stent exhibited a significantly lower propensity for migration compared to the straight‐type biliary plastic stent (OR = 2.654, *p* = 0.034). In addition, the risk of proximal biliary stent migration increased significantly with the degree of balloon dilation (OR = 2.708, *p* = 0.043) and the presence of diverticula in the descending part of the duodenum (OR = 6.412, *p* = 0.002).

**Conclusion:** Duodenal diverticulum, duodenal sphincterotomy, longer biliary stents, and straight biliary stents are independent risk factors for stent migration. In addition, balloon dilation greater than 1 cm and the presence of diverticula in the descending part of the duodenum are independent risk factors for proximal biliary stent migration. Consequently, patients with risk factors for stent migration should be closely monitored.

## 1. Introduction

Choledocholithiasis is a common and frequently occurring condition of the digestive system. Endoscopic retrograde cholangiopancreatography (ERCP) is the preferred approach for treating common bile duct (CBD) stones, regardless of the patient’s age [1]. Endoscopic biliary plastic stents have been widely employed for stable biliary drainage following ERCP, with a success rate approaching 100% [2].

While biliary plastic stent implantation offers several advantages in the early postoperative period, including effective drainage and low rates of adverse events, prolonged retention of these stents can lead to a series of complications such as stent migration (proximal/distal), stent fragmentation, occlusion, recurrent choledocholithiasis, and acute cholangitis. Therefore, the removal of these stents within 1–3 months postoperatively is recommended [3]. Although distal migration of the biliary stent from the CBD has been reported occasionally, most detached stents are excreted from the body through intestinal peristalsis. However, a few studies have indicated that detached biliary stents can cause colonic perforation [4]. Moreover, plastic stents that displace into the biliary tract may induce recurrent biliary obstruction or infection, often necessitating repeat ERCP to remove the residual stent and cleanse the biliary tract. This situation can increase the financial burden on patients and heighten the risk of postoperative adverse events.

The current literature regarding the migration of biliary plastic stents is limited. Consequently, this study aimed to retrospectively analyze the clinical data of patients who underwent ERCP for cholelithiasis at our center, with the objective of exploring the incidence and risk factors associated with migration of biliary plastic stents following bile duct stone removal during ERCP.

## 2. Materials and Methods

### 2.1. Patients

This study collected relevant data from 2527 patients diagnosed with choledocholithiasis accompanied by acute cholangitis who underwent ERCP stone removal at the Digestive Endoscopy Center of the 900th Hospital of the Joint Logistics Support Force between March 2018 and December 2022. The patients were divided into two groups based on the drainage methods after ERCP stone removal: endoscopic nasobiliary drainage (ENBD) (1635 cases) and endoscopic biliary stent placement (892 cases), and the clinical data of 892 patients were retrospectively analyzed.

The inclusion criteria were as follows:

1. Patients aged 18–90 years who were diagnosed with choledocholithiasis based on preoperative abdominal B‐ultrasonography, computed tomography, magnetic resonance cholangiopancreatography, or ultrasonic gastroscopy, and who subsequently underwent bile duct stone removal via ERCP.
2. Patients who received a temporary biliary plastic stent for internal drainage after ERCP.

The exclusion criteria were as follows:

1. Patients with a history of major pancreaticobiliary resection (defined as pancreaticoduodenectomy, distal pancreatectomy, total pancreatectomy, or hepatectomy involving biliary confluence).
2. Cholangiolithiasis that could not be removed with ERCP and required long‐term implantation of a plastic stent.
3. Choledocholithiasis secondary to biliary pancreatic malignancy.
4. Patients who failed to return to the hospital within the prescribed time frame to remove the biliary stent.
5. Patients with relevant clinical data that were missing or lost to follow‐up.

**Figure 1:**
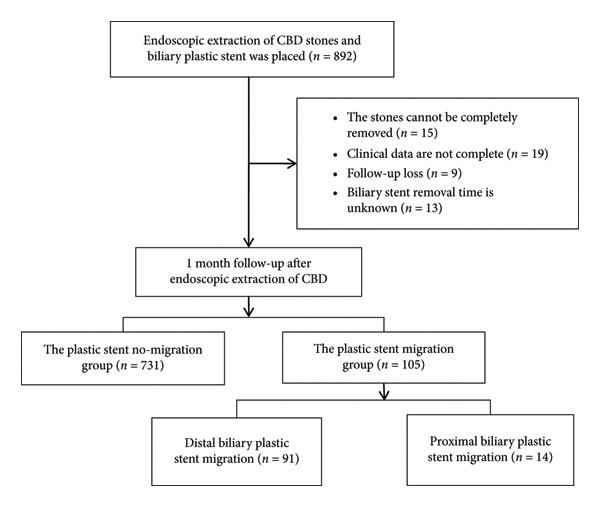
Flowchart outlining the study protocol.

We collected data from 836 eligible patients on demographic (sex, age, and body mass index), history of cholecystectomy and gastrectomy, endoscopic papillary sphincterotomy (endoscopic sphincterotomy [EST]), stent type (model, length, and size), bile duct angle, postoperative adverse events, and other relevant data of all eligible patients (Figure 1). This study was approved by the Ethics Committee of the 900th Hospital of the People’s Liberation Army (PLA) (no. 2018XHNK03). Patients and their families were informed of the potential risks and benefits of participation and signed informed consent forms.

### 2.2. Endoscopic System

The procedures performed in this study utilized a Japanese Olympus TJF‐260 electronic duodenoscope or Pentax ED‐341C, along with a German ERBE high‐frequency electric cutting device, a Boston yellow zebra guidewire, a needle knife fistulotomy catheter, a stone extraction balloon, a stone–gravel integrated basket, and a straight‐type plastic stent with center bend (Advanix Biliary Stent; NaviFlex, Boston, USA), and a Christmas tree‐shaped stent, as well as snares.

### 2.3. ERCP Procedure

ERCP was performed by 2 expert endoscopists who had more than 3 years of experience with advanced endoscopy, and the number of cases is greater than 1000. The procedure was carried out using a standard duodenoscope, and biliary cannulation was initially attempted with a wire‐guided catheter, after selectively cannulating the bile duct and obtaining cholangiogram; the decision to perform either EST or endoscopic papillary balloon dilation (EPBD) is based on the bile duct diameter, along with the number and size of the stones (EST: preferred for stones ≥ 10 mm and CBD diameter ≥ 12 mm) or concomitant biliary strictures. The appropriate length of EST should be adjusted according to the papillary anatomy and stone size (EPBD: preferred for stones < 8 mm, CBD diameter < 12 mm, and coagulopathy [INR > 1.5]), or younger patients who intend to preserve sphincter function. If needed, mechanical lithotripsy may be conducted. Balloon occlusion cholangiography was performed by injecting contrast, and fluoroscopic assessment using X‐ray was applied to confirm the complete removal of CBD stones. At the end of the procedure, for patients with specific conditions, such as preoperative cholangitis, intraoperative papillary edema, hemorrhage, and the need for lithotripsy, ENBD or biliary stenting was performed if necessary. Postoperatively, measures were taken to ensure fasting and inhibition of pancreatic secretion, along with fluid rehydration and additional treatments as necessary. Close monitoring of the patients’ vital signs, routine blood parameters, blood amylase levels, C‐reactive protein (CRP) levels, and other clinical indices was conducted at 3 and 24 h post‐ERCP.

### 2.4. Follow‐Up Procedure

Abdominal plain radiography was conducted within 1 month after ERCP to assess the position of the biliary plastic stent. If the biliary stent was properly positioned, snare removal under gastroscopy was deemed feasible. Conversely, if the biliary stent was migrated into the intrahepatic bile duct or the middle and upper segments of the CBD, a stone extraction balloon combined with a snare was utilized to retrieve the stent under ERCP guidance. Patients underwent telephone follow‐up assessments postoperatively, during which abdominal pain, jaundice, fever, and other related symptoms were documented. If necessary, routine blood evaluations, including measurements of blood amylase, CRP, and other indicators, were performed to assess the presence of acute cholangitis or acute pancreatitis. If the stent had fallen into the intestinal cavity, abdominal plain radiographs were regularly obtained to monitor changes in the stent’s position until it was expelled from the intestinal tract. Throughout this period, the onset of abdominal pain and fever was closely monitored.

### 2.5. Adverse Events After ERCP

This study monitored the following adverse events following ERCP: (1) Post‐ERCP hyperamylasemia, which is defined as elevated blood amylase levels exceeding three times the normal value without any obvious clinical symptoms 24 h after ERCP; (2) post‐ERCP pancreatitis (PEP), which was diagnosed when newly onset or worsening abdominal pain emerged that was associated with an elevation in serum amylase of at least 3 times the normal range 24 h after ERCP; and (3) ERCP‐related bleeding, which is indicated by hematemesis, melena, or other signs during the procedure, accompanied by a decrease in hemoglobin levels of ≥ 30 g/L.

### 2.6. Migration of Biliary Plastic Stents

**Figure 2:**
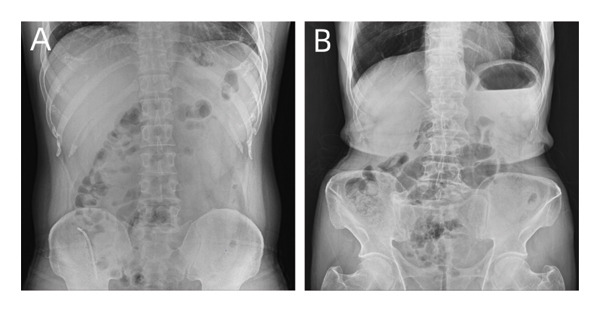
Migration of biliary plastic stents. (A) The biliary plastic stent falls off into the intestine. (B) The biliary plastic stent was moved inside the bile duct.

Migration of biliary plastic stents was classified into the following categories: (1) Distal biliary plastic stent migration (DSM): DSM was defined if the distal tip of the stent was no longer present at the papillary orifice nor fuoroscopically visible in the bile duct on follow‐up endoscopic and fluoroscopic examination (Figure 2(A)) and (2) proximal biliary plastic stent migration (PSM): PSM was defined if the distal tip of the stent was no longer visible at the ampulla, but visible on fluoroscopy to be in the bile duct (Figure 2(B)).

### 2.7. Statistical Analysis

Data were collected and analyzed using the Statistical Package for the Social Sciences (SPSS) Version 25.0. Measurement data demonstrating a normal distribution are expressed as mean ± standard deviation, while median (P25 and P75) is used for data exhibiting a non‐normal distribution. Count data are reported as a number (%). Differences between groups were evaluated using the *t*‐test or the Mann–Whitney U test. Count data are expressed as constituent ratios or rates (%), and the chi‐square test was utilized to compare differences between the groups. Univariate analysis was performed for variables potentially associated with the migration of biliary plastic stents, followed by logistic regression analysis, with a *p* value of < 0.05 indicating statistical significance.

## 3. Results

During the study period, a total of 836 patients (511 males and 325 females; mean age: 60.40 ± 15.58 years) were ultimately included in the analysis. The migration group comprised 105 patients, including 91 who experienced DSM and 14 who exhibited PSM. All stents that were shed were expelled spontaneously from the intestine, whereas those demonstrating proximal migration into the CBD were subsequently removed via ERCP. The specific demographic characteristics of the patients are presented in Table 1.

**Table 1:** Clinical features in patients showing migration and no migration of the biliary plastic stent after stone removal with endoscopic retrograde cholangiopancreatography.

Characteristics	No‐migration group (*n* = 731)	Migration group (*n* = 105)	*p* value
Sex, male/female	447/284	64/41	0.897
Age (years)	60.51 ± 15.77	59.63 ± 14.27	0.586
BMI (kg/m^2^)	23.13 (21.05, 25.11)	23.44 (21.83, 25.41)	0.231
Follow‐up duration (days)	29 (27, 33)	30 (28,36)	0.072
EST	103 (14.1)	5 (4.8)	0.008
EPBD			0.251
1.0–1.5 cm	177 (24.2)	38 (36.2)	
0–1.0 cm	439 (60.1)	45 (42.9)	
Undilated	115 (15.7)	22 (20.9)	
Pancreatic duct stent	41 (5.6)	8 (7.6)	0.412
Angulation of the bile duct (> 145°)	43 (5.9)	10 (9.5)	0.152
Biliary stent length (cm)			< 0.001
< 7	348 (47.6)	70 (66.7)	
≥ 7	383 (52.4)	35 (33.3)	
Duodenal diverticula	56 (7.7)	17 (16.2)	0.004
Cholecystectomy	227 (31.1)	30 (28.6)	0.606
Postgastrectomy	13 (1.8)	3 (2.9)	0.708
Biliary stent type			0.032
Christmas tree‐shaped	76 (10.4)	4 (3.8)	
Straight‐type biliary plastic stent	655 (89.6)	101 (96.2)	
Size of the common bile duct (mm)			0.404
< 8	331 (45.3)	43 (41.0)	
≥ 8	400 (54.7)	62 (59.0)	
Plastic stent size			0.016
7‐Fr	532 (72.8)	88 (83.8)	
8.5‐Fr	199 (27.2)	17 (16.2)	

Abbreviations: BMI, body mass index; EPBD, endoscopic papillary balloon dilatation; EST, endoscopic sphincterotomy.

Based on the results presented in Table 1 and guided by clinical judgment, we identified several potential factors associated with the migration of biliary plastic stents following ERCP for stone removal: papillary sphincter incision, plastic stent size, stent length, and the presence of diverticula. Utilizing these factors as independent variables, we conducted a multifactor logistic regression analysis with the occurrence of migration as the dependent variable. The results, detailed in Table 2, indicated that both stent length and type, duodenal sphincter incision, and the presence of diverticula were independent risk factors for biliary plastic stent migration.

**Table 2:** Logistic regression analyses of the risk factors for biliary plastic stent migration after stone removal with endoscopic retrograde cholangiopancreatography.

Risk factors	OR (95% CI)	*p* value
EST	3.638 (1.435–9.255)	0.007
Duodenal diverticula	2.367 (1.300–4.307)	0.005
Biliary stent type (Christmas tree‐shaped stent)	2.654 (1.002–5.112)	0.034
Biliary stent length (≥ 7 cm)	0.423 (0.273–0.655)	< 0.001
Plastic stent size	0.789 (0.432–1.443)	0.442

Abbreviations: CI, confidence interval; EST, endoscopic sphincterotomy; OR, odds ratio.

DSM occurred in 91 patients. During the follow‐up period, all of these stents were spontaneously excreted through intestinal peristalsis, with no incidents of intestinal perforation or stent retention. In the group that did not demonstrate stent migration, the biliary plastic stents were removed under gastroscopic guidance approximately 1 month later, with no adverse effects recorded during the procedure. Stent migration to the middle and upper portions of the CBD was noted in 14 patients, representing 13.3% of the cases involving stent migration. All displaced stents were subsequently removed using ERCP, with no adverse events reported in these patients post‐ERCP.

Based on the results presented in Table 3 and our professional judgment, we identified the factors associated with proximal migration of the biliary stent: age, the status of biliary balloon dilatation, and the presence of diverticula. Utilizing these factors as independent variables and proximal migration as the dependent variable, we performed a multivariate logistic regression analysis. The results indicated that the degree of balloon dilatation and the presence of diverticula were independent risk factors for PSM, as detailed in Table 4.

**Table 3:** Comparison of clinical features between patients showing no‐PSM group and those showing PSM group after stone removal with endoscopic retrograde cholangiopancreatography.

Characteristics	No‐PSM group (*n* = 822)	PSM group (*n* = 14)	*p* value
Sex, male/female	501/321	10/4	0.425
Age (years)	60.26 ± 15.56	69.07 ± 14.77	0.036
BMI (kg/m^2^)	23.36 (21.33, 25.15)	23.10 (20.62, 23.93)	0.370
Follow‐up duration (days)	30 (27, 33)	33 (28, 36)	0.096
EST	108 (13.1)	0 (0.0)	0.146
EPBD			0.005
1.0–1.5 cm	207 (25.2)	8 (57.1)	
0–1.0 cm	478 (58.1)	6 (42.9)	
Undilated	137 (16.7)	0 (0.0)	
Pancreatic duct stent	47 (5.7)	2 (14.3)	0.195
Angulation of the bile duct (> 145°)	52 (6.3)	1 (7.1)	0.603
Biliary stent length (cm)			0.946
< 7	410 (49.9)	8 (57.1)	
≥ 7	412 (50.1)	6 (42.9)	
Duodenal diverticula	68 (8.3)	5 (35.7)	0.002
Cholecystectomy	254 (30.9)	3 (21.4)	0.446
Postgastrectomy	15 (1.8)	1 (7.1)	0.238
Biliary stent type			0.247
Christmas tree‐shaped stent	79 (9.6)	1 (7.1)	
Straight‐type biliary plastic stent	743 (90.4)	13 (92.9)	
Size of the common bile duct (mm)			0.220
< 8	370 (45.0)	4 (28.6)	
≥ 8	452 (55.0)	10 (71.4)	
Plastic stent size			0.704
7‐Fr	609 (74.2)	11 (78.6)	
8.5‐Fr	213 (25.9)	3 (21.4)	

Abbreviations: BMI, body mass index; EPBD, endoscopic papillary balloon dilatation; EST, endoscopic sphincterotomy.

**Table 4:** Logistic regression analyses of the risk factors for proximal biliary plastic stent migration after stone removal with endoscopic retrograde cholangiopancreatography.

Risk factors	OR (95% CI)	*p* value
Age	1.032 (0.988–1.078)	0.157
Duodenal diverticulum	6.412 (2.010–20.451)	0.002
EPBD (> 1 cm)	2.708 (1.196–3.243)	0.043

Abbreviations: CI, confidence interval; EPBD, endoscopic papillary balloon dilatation; OR, odds ratio.

All enrolled patients successfully underwent bile duct stone removal via ERCP in a single procedure. The incidence of postoperative adverse events, including pancreatitis, biliary tract infection, and biliary tract hemorrhage, did not differ significantly between the migration group and the no‐migration group, as indicated in Table 5.

**Table 5:** Occurrence of complications in the plastic stent migration and no‐migration groups after stone removal with endoscopic retrograde cholangiopancreatography.

Complications	No‐migration group (*n* = 731)	Migration group (*n* = 105)	*p* value
	166/565 (29.4%)	20/85 (23.5%)	0.399

## 4. Discussion

Biliary stones are the leading cause of hospitalization for gastrointestinal diseases worldwide [5]. In the United States, approximately 20–25 million individuals (10%–15% of the adult population) are affected by gallstones [6], which represent a significant portion of medical expenses, totaling $4.3 billion [7]. The incidence of cholelithiasis in China, Japan, South Korea, and other Asian countries is notably higher than in Western nations, attributed to recent improvements in living standards and changes in dietary habits, with rates continuing to rise in these regions [8].

Advancements in endoscopic minimally invasive techniques and associated instruments have established ERCP combined with sphincterotomy and stone removal as the preferred treatment for CBD stones. ERCP can successfully remove CBD stones in up to 90% of patients, offering advantages such as less trauma [9], reduced adverse events, and rapid postoperative recovery. However, incisions of the papillary sphincter, balloon compression, and repeated stone stimulation during the procedure can lead to significant edema of the mucosa surrounding the papilla, potentially inducing acute cholangitis and pancreatitis. Consequently, to ensure stable postoperative drainage of bile and pancreatic juice, ENBD or endoscopic biliary plastic stent placement (ERBD) is often performed. Both methods exhibit unique advantages and disadvantages for bile drainage, with no significant differences in efficacy or adverse events reported [10] ENBD, while effective, may result in considerable bile loss and discomfort postoperation. ERBD, as an internal drainage technique, is widely used in clinical practice for biliary drainage due to its advantages of low cost and simple operation. This technique is characterized by no bile and electrolyte loss, no postoperative discomfort, no impact on the patient’s appearance, and the ability to remain for a long time [11], hence more and more patients tend to choose biliary plastic stent placement. In our center, for patients with the following conditions, such as preoperative cholangitis, multiple mechanical lithotripsy in the bile duct, papillary bleeding and significant swelling caused by prolonged operation, and strong patient willingness, we tend to place biliary stents for short‐term drainage after successful stone removal by ERCP. Furthermore, Choi et al. reported that the placement of biliary plastic stents not only accelerates recovery from acute cholangitis caused by CBD stones but also helps reduce the recurrence rate of bile duct stones in patients undergoing ERCP stone fragmentation treatment [12].

The use of biliary stents in the management of biliary and pancreatic diseases has been increasing; however, the associated risk of stent migration is also rising. In our cohort, stent migration occurred in 12.6% of cases, higher than the previously reported migration rate for plastic stents in 5%–10% [13, 14]. This discrepancy may be attributed to the composition of our study population, which primarily included patients with bile duct stones and excluded those with benign or malignant biliary strictures. The mechanism of stent migration may involve the following factors: (1) Significant dilation of the bile duct and large balloon dilation at the papilla may affect the frictional force between the stent and the bile duct wall, weakening the anchoring effect of the stent and increasing the risk of migration [15]. (2) Postsphincterotomy or the presence of periampullary diverticula may impair the contractile function of the papillary sphincter, leading to a relaxed state of the Oddi sphincter, which affects the fixation around the stent. In addition, repeated flushing during bile excretion can easily cause stent migration [16]. (3) Resolution of preexisting biliary inflammation, edema, or stenosis may eliminate the stable anchoring structure of the stent, increasing its mobility [17]. (4) Due to the inconsistent physical characteristics of different stents, there are varying radial forces and antimigration anchoring flanges. The smaller the radial force, the more likely the stent is to migrate [15].

Research has indicated that migration of biliary stents can occur, and in rare cases, stents migrating into the intestine may lead to intestinal perforation, associated with intestinal structural abnormalities such as postoperative adhesions, colonic diverticula, and hernias [18, 19]. However, the factors influencing biliary stent migration from the CBD to the upper segments of the CBD and distal intestine are not extensively studied in the current literature. Our study identified the incision of the duodenal papillary sphincter, the presence of diverticula at the papilla, and the length and type of biliary stent as independent risk factors for migration of biliary plastic stents. Patients with CBD stones exhibiting these characteristics are at a heightened risk for stent migration and related adverse events during ERCP and postoperative follow‐up. The onset of abdominal pain should be regarded as a warning signal necessitating caution and preventive measures against adverse outcomes. Previous studies [20] have demonstrated that the likelihood of intestinal perforation escalates with long stents or those shaped like Christmas trees, thus, careful consideration should be given when selecting such stents. For high‐risk patients, nasobiliary drainage may be advisable during ERCP, or patients should be instructed to return promptly for biliary plastic stent removal when necessary. Our study found that while 91 patients experienced migration of biliary plastic stents into the intestinal cavity during follow‐up, all these stents were successfully expelled without significant adverse reactions.

In addition, 14 patients in our study exhibited biliary plastic stent migration. The PSM rate in our study was significantly lower than the 6.5% reported by Kale et al., which is due to the fact that the duration of stent placement in our study rarely exceeded 3 months [21]. Besides acute cholangitis and stent obstruction caused by stent retention in the bile duct, adherence of bile sludge to the stent may lead to the reformation of bile duct stones. In severe cases, it may lead to perforation of intrahepatic structures [22]. The findings indicate that diverticula at the papilla and the degree of balloon dilation are independent risk factors for proximal migration of biliary plastic stents, where abnormal structures around the duodenal papilla further increase the risk of stent migration. Therefore, for patients with a diverticulum at the descending duodenal papilla or those requiring large balloon dilation of the distal CBD for stone extraction, if a biliary plastic stent is placed postoperatively, it is recommended that the patient return for endoscopic stent removal 2–3 weeks later. If abdominal pain occurs during this period, a prompt abdominal CT scan should be performed to assess the condition of the stent.

This study acknowledges certain limitations. As a single‐center retrospective study, it was not possible to accurately control the timing of biliary stent removal in the outpatient department, which may have impacted the assessment of stent migration. Second, because the biliary plastic stents used at our hospital are straight‐type plastic stents with a center bend, they present a higher risk of migration compared to plastic stents with double pig tail [23]. We plan to conduct a multicenter, large‐sample prospective controlled study to further evaluate the migration risks associated with different types of stents.

Migration of plastic biliary stents frequently occurs during ERCP treatment. Therefore, for patients with a duodenal papillary diverticulum requiring large balloon dilation during stone removal via ERCP, nasobiliary stent placement should be considered to avoid PSM and to optimize patient outcomes.

**Nomenclature**
ERCP: Endoscopic retrograde cholangiopancreatography
ERBD: Endoscopic biliary plastic stent placement
ENBD: Endoscopic nasobiliary drainage
EPBD: Endoscopic papillary balloon dilatation
EST: Endoscopic sphincterotomy
CBD: Common bile duct
CT: Computed tomography
MRCP: Magnetic resonance cholangiopancreatography
CRP: C‐reactive protein

## Ethics Statement

This study was approved by the Ethics Committee of the 900TH Hospital of PLA. All methods of this study were performed in accordance with the Declaration of Helsinki.

## Disclosure

This article has been published as a preprint [24].

## Conflicts of Interest

The authors declare no conflicts of interest.

## Author Contributions

Longping Chen, Yanfang Lin, Linfu Zheng, Chuanshen Jiang, Zhiping Chen, Dazhou Li, and Wen Wang participated in the acquisition of data. Longping Chen, Yanfang Lin, and Linfu Zheng contributed to the manuscript drafting. Longping Chen, Jin Zheng, Chuanshen Jiang, Zhiping Chen, Dazhou Li, and Wen Wang contributed to the analysis and interpretation of data, as well as revision of the manuscript for important intellectual content. Longping Chen, Yanfang Lin, and Linfu Zheng contributed equally to this project and should be regarded as co‐first authors.

## Funding

This project was supported by the Fujian Science and Technology Guiding Fund Project (Grant no. 2023Y0070), the Foreign Cooperation Project of the Department of Science and Technology of Fujian Province (Grant no. 2019I0026), and the Key Subject Funds of Joint Logistics Support Force (Grant no. LQZD‐XH).

## Data Availability

The data that support the findings of this study are available on request from the corresponding author. The data are not publicly available due to privacy or ethical restrictions.
